# Initial Presentation of Cirrhosis Mimicking an Ischemic Stroke

**DOI:** 10.7759/cureus.19474

**Published:** 2021-11-11

**Authors:** Navkiran K Randhawa, Toral Shastri, Es-Haq Hassanin, Sukhbir Randhawa

**Affiliations:** 1 Internal Medicine, Franciscan Health, Olympia Fields, USA; 2 Internal Medicine, Midwestern University Chicago College of Osteopathic Medicine, Downers Grove, USA; 3 Internal Medicine, University of Texas Health Science Center at Tyler, Tyler, USA; 4 Internal medicine, Samaritan Medical Center, Watertown, USA

**Keywords:** stroke mimic, transaminitis, hyperammonemia, hepatic encephalopathy, cirrhosis

## Abstract

Liver cirrhosis is the 12th most common cause of death in the United States of America. This disease commonly presents with neurological disorders including, but not limited to, dementia, asterixis or coma. Hepatic encephalopathy and hyperammonemia are suspected to be the cause of altered mentation. However, hepatic encephalopathy with neurological symptoms mimicking stroke is underestimated. We present a case of liver cirrhosis manifesting with initial signs of right-sided hemiparesis.

## Introduction

Liver cirrhosis is a chronic liver disease that causes degeneration and necrosis of hepatocytes as well as replacement of the liver parenchyma by fibrotic tissues and regenerative nodules [1]. In the United States (US), an estimated 44,000 deaths per year are attributed to chronic liver disease and cirrhosis [2]. Blacks and Hispanics have the higher prevalence of cirrhosis in the US in addition to those with lower levels of education [2]. Unfortunately, many cases often go undiagnosed and 69% of people in the United States are unaware of having liver disease [3]. Cirrhosis can present once complications develop, including variceal hemorrhage, ascites, spontaneous bacterial peritonitis, and hepatic encephalopathy [4]. Hepatic encephalopathy results from an elevation in blood ammonia and compromises 30%-45% of complications developing from cirrhosis [5]. It is often easy to detect in patients with overt neuropsychiatric symptoms associated with well-known presentations such as sleep pattern changes, asterixis, hyperactive deep tendon reflexes, and transient decerebrate posturing in rare cases [4]. Hepatic disease is rarely considered in patients presenting with focal neurological deficits associated with a specific vascular distribution [6].

## Case presentation

A 54-year-old, Hispanic male with no known past medical history presented to the emergency room with a chief complaint of sudden-onset right-sided weakness over the past 24 hours. The patient denied any associated dizziness, visual disturbance, or reduced level of consciousness. He had not seen a physician in over 20 years. His social history was significant for chronic alcohol abuse with consumption of at least one pint of tequila or beer per day for at least 35 years. The patient was tachycardic to 101/min with a physical exam that was remarkable for right-sided facial droop as well as 1/5 strength in the right upper and lower extremities. He was noted to have a National Institutes of Health stroke scale of 17.

**Figure 1:**
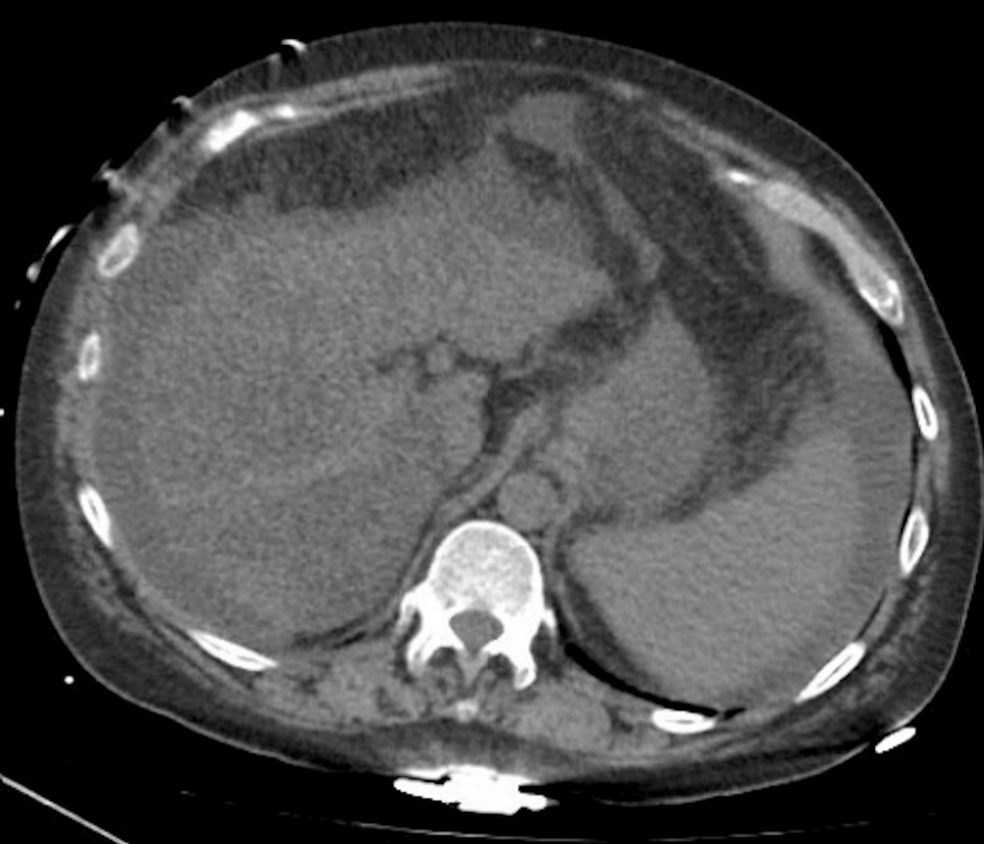
Micronodular appearance of the liver with numerous scattered hypodensities

A stat computed tomography (CT) of the head without contrast as well as magnetic resonance imaging (MRI) without contrast were negative for acute stroke. The patient's urine drug screen and alcohol level was unremarkable. His labs were significant for an ALT of 70, AST 251, direct bilirubin 0.4, INR 1.1, platelets 79 x 10^3 (Table 1). All of his other labs were normal. Further investigation with right upper quadrant ultrasound suggested possible liver cirrhosis. Computed tomography of the abdomen and pelvis revealed a micronodular appearance of the liver with numerous scattered hypodensities (Figure 1). A hepatitis A, B, C panel, autoimmune panel, alpha-1 antitrypsin deficiency panel and iron panel were all negative. Our patient was suspected to have alcohol-induced cirrhosis due to his extensive alcohol history.

**Table 1:** Initial abnormal venous laboratory values and follow-up values

	Before admission	Admission	36 hr after admission	1-month hospital follow-up
ALT	N/A	70	60	52
AST	N/A	251	220	62
Direct bilirubin	N/A	0.4	0.4	0.2
Ammonia	N/A	140	N/A	38
INR	N/A	1.1	1.1	1.0
Platelets	N/A	79	83	87

A lipid panel and Doppler of the neck were obtained which were found to be normal. Once an ammonia level was found to be 140 micro mol/L, the patient was started on lactulose and rifaximin. The patient opted out of physical therapy the first two days of admission; however, his neurological symptoms resolved without residual neurological deficits about 36 hours after treatment of lactulose and rifaximin. We discharged our patient with close follow-up at a GI clinic and repeat labs one month after discharge (Table 1).

## Discussion

Hepatic encephalopathy secondary to cirrhosis is associated with cognitive and neuromuscular impairment [4]. Focal neurologic deficits and unilateral weakness, however, are typically associated with acute cerebrovascular accidents. Prior reports have described a presentation of hemiparesis with a normal initial and repeated CT with contrast [7]. In these studies, the initial T2-weighted MRI of the brain showed a high-intensity lesion of 5-6 cm in the right frontal lobe and multiple lesions in both parietal and occipital lobes as a result of hypoperfusion [7]. It was later hypothesized that the neurotoxicity and neurotransmitter derangements of hepatic encephalopathy may have created focal neurologic signs due to diminished perfusion and impaired neurotransmitter function within the subclinical stable lesions [7]. Another case was reported of a patient with hepatic encephalopathy and transaminitis experiencing new-onset expressive aphasia, left gaze deviation, and right-sided hemiparesis [6]. The patient’s CT revealed multiple subcortical patchy hypodensities and FLAIR revealed chronic microvascular findings bilaterally [6]. After normalization of liver enzymes and treatment with lactulose, neurological deficits resolved [6]. Another study with prospectively collected data showed 6 out of 46 (13%) hospitalized patients with hepatic encephalopathy initially presented with hemiplegia or hemiparesis [8]. None of these patients showed significant initial or follow-up CT or MRI findings and symptoms were fully reversed upon regression of the hepatic encephalopathy [8].

Our presentation depicts a Hispanic male presenting to the ER with a right-sided facial droop and hemiparesis. During CT and MRI imaging, the patient did not have any evidence of acute ischemic stroke or hypodense lesions. However, the patient’s ammonia levels were twofold higher than the upper limit of normal and normalized one month after discharge. We predict the patient’s neurological symptoms in the absence of vascular compromise occurred due to a metabolic demand failure and neurotransmitter derangements in the setting of hyperammonemia. However, further studies are warranted to determine what makes a specific hemisphere more susceptible to effects in the absence of microvascular changes. This case also highlights the importance of considering nonischemic causes in patients presenting to the hospital for stroke-like symptoms. In patients presenting with stroke-like symptoms and no available medical history, measuring ammonia levels, liver enzymes, and coagulation studies during routine blood work may be of value.

## Conclusions

Cirrhosis is generally asymptomatic in around 40% of patients and is usually diagnosed during routine examinations. Cirrhosis manifesting with acute stroke-like symptoms is an underestimated entity. Our study highlights the importance of considering non-neurological pathologies in the setting of acute hemiparesis. The gastrointestinal system is often overlooked when it comes to these symptoms. We encourage initial evaluation with liver enzymes and further workup is indicated if a patient with hemiparesis is negative for acute stroke.
